# P-1907. Comparing Multisystem Inflammatory Syndrome in Children (MIS-C) Case Characteristics Across Periods of SARS-CoV-2 (SC2) Variant Predominance in Los Angeles County (LAC): 2020-2023

**DOI:** 10.1093/ofid/ofae631.2068

**Published:** 2025-01-29

**Authors:** Christina Collins, Annabelle de St Maurice, Elizabeth Traub, Israa Khanqadri, Lauren E Finn

**Affiliations:** Los Angeles County Department of Public Health, Los Angeles, California; University of California Los Angeles, Los Angeles, California; Los Angeles County Department of Public Health, Los Angeles, California; Los Angeles County Department of Public Health, Los Angeles, California; Los Angeles County Department of Public Health, Los Angeles, California

## Abstract

**Background:**

MIS-C is a rare condition in children associated with prior SC2 infection. Limited data show the impact of SC2 variants on the presentation of MIS-C. This study analyzes differences in the presentation of MIS-C cases in LAC when different variants were circulating.

Characteristics of Multisystem Inflammatory Syndrome in Children (MIS-C) Cases, -- Los Angeles County*, California March 1, 2020- December 31, 2023

**Figure:**
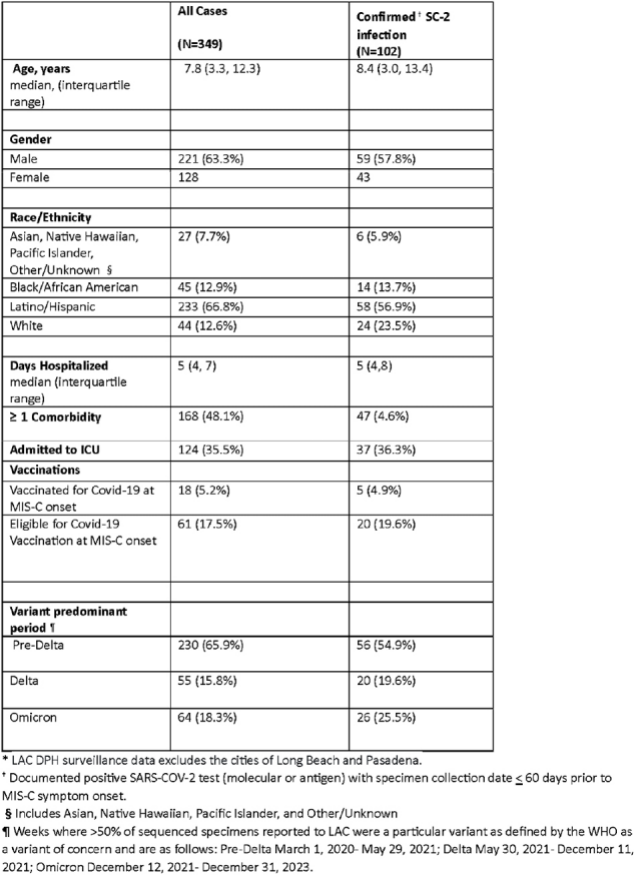

**Methods:**

This analysis includes MIS-C cases in people < 21 years of age reported to LAC with onset from 3/1/2020-12/31/2023. We defined confirmed SC2 infection as having a documented positive SC2 test with specimen collection date < 60 days prior to MIS-C symptom onset. Where SC2 positive results were not available, we imputed SC2 infection as occurring 30 days prior to MIS-C onset. Infection dates were used to classify cases by variant predominant periods, defined as weeks where > 50% of sequenced specimens reported to LAC were a particular variant as defined by the WHO as a variant of concern. Case demographics and clinical characteristics were compared across variant periods. Analyses were conducted for all reported MIS-C cases and for cases with confirmed SC2 infection. Statistical significance was tested with the Kruskal-Wallis test and chi-squared test [α=0.05].

**Figure:**
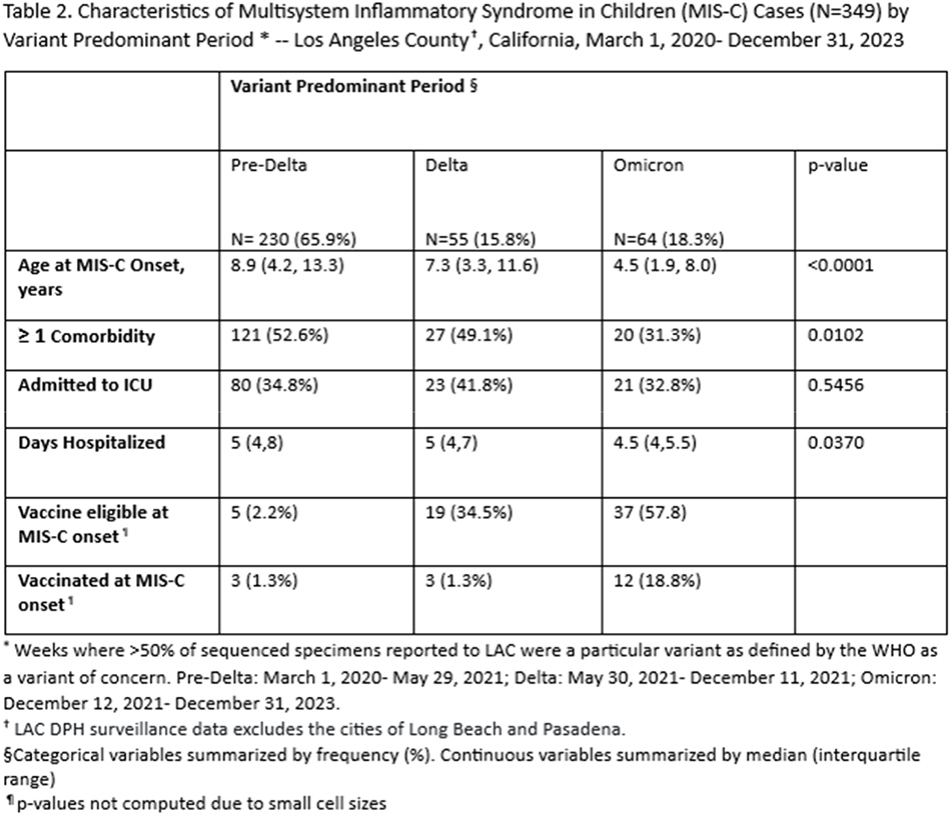

**Results:**

349 MIS-C cases were reported between 2020-2023 (Table 1). Of these, 102 (29.2%) had a documented SC2 infection. Median age at onset was 7.8 years. 168 (48.1%) had ≥ 1 comorbidity, 124 (35.5%) were admitted to the ICU, and median hospital length of stay (LOS) was 5 days. 18 cases were vaccinated prior to MIS-C onset. The most SC2 infections occurred during the Pre-Delta period (230, 65.9%), while the fewest occurred during the Delta period (57, 16.3%) (Table 2). Median age and proportion of children with ≥ 1 comorbidity decreased across variant periods (p < 0.0001, p=0.0102; respectively). The median LOS was lowest when SC2 infection occurred during the Omicron period (p=0.0370). ICU admission was not statistically different across variant periods.

**Figure:**
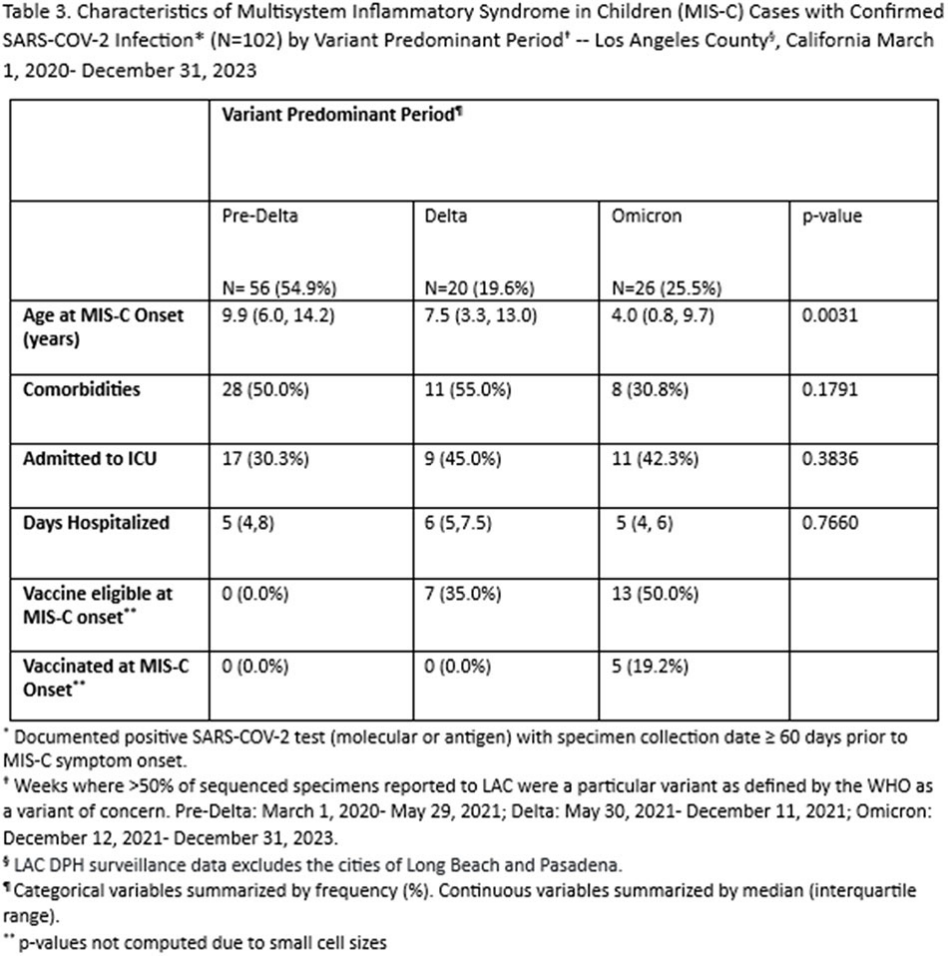

**Conclusion:**

The LOS, median age, and proportion of cases with comorbidities decreased when SC2 infection occurred during the Omicron period. This may be due to differences in variant pathogenicity, natural immunity decreasing the MIS-C susceptible pool, and younger children being the last group to be eligible for vaccination during later variant periods.

**Figure:**
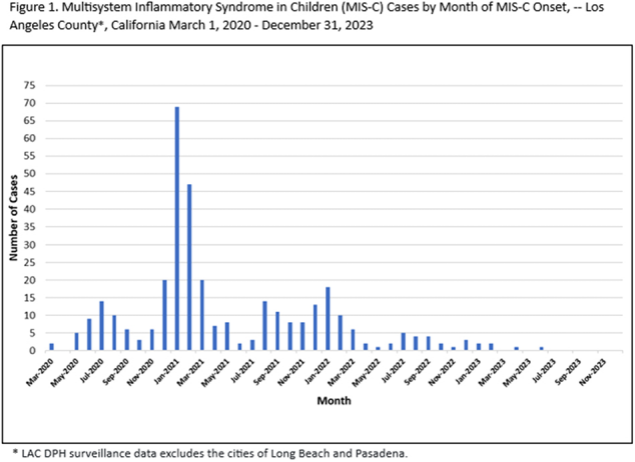

**Disclosures:**

All Authors: No reported disclosures

